# Perinatal post mortem ultrasound (PMUS): a practical approach

**DOI:** 10.1186/s13244-019-0723-9

**Published:** 2019-03-18

**Authors:** Susan C. Shelmerdine, Neil J. Sebire, Owen J. Arthurs

**Affiliations:** 1 Great Ormond Street Hospital for Children NHS Foundation Trust, London, WC1N 3JH UK; 2 UCL Great Ormond Street Institute of Child Health, London, UK

**Keywords:** Ultrasound, Autopsy, Diagnostic imaging, Child, Methodology

## Abstract

Declining rates of consent for standard perinatal autopsy has led to a rise in interest for postmortem imaging as an alternative, non-invasive method for investigation of childhood and perinatal deaths. Whilst much interest has focussed on cross-sectional techniques such as postmortem CT (PMCT) or MRI (PMMR), other modalities including postmortem ultrasound (PMUS) have been shown to have reasonable diagnostic accuracy rates, with the added benefit of being more readily accessible and affordable. There is little published information or formal guidance available on preparation for postmortem perinatal ultrasound, views to be obtained and differentiating normal postmortem change from potential abnormalities.

This article will focus on the role of perinatal postmortem ultrasound as an alternative imaging method for non-invasive autopsy, with emphasis on imaging technique, practical considerations and commonly encountered case examples.

**Electronic supplementary material:**

The online version of this article (10.1186/s13244-019-0723-9) contains supplementary material, which is available to authorized users.

## Key points

- Non-invasive imaging offers a solution for parents declining conventional autopsy.
- Postmortem ultrasound can delineate main body organs and identify congenital anomalies.
- Postmortem ultrasound can be useful where access to MRI is unavailable.
- Postmortem ultrasound may aid image-guided organ biopsies in the future.

## Background

The chronic decline in parental consent rates for perinatal autopsies has contributed to the rise of non-invasive methods for death investigation [1]. Traditionally, whole body radiography (i.e. skeletal surveys) has been the mainstay of postmortem imaging; however, their usage is limited outside detecting inheritable bone diseases [2]. Large cohort studies have investigated the use of postmortem CT (PMCT) [3, 4] and MRI [5–7], with better concordance seen in perinatal postmortem MRI (PMMR) of up to 90% when compared to autopsy [6]. Unfortunately, both PMCT and PMMR can be costly and difficult to access, thereby limiting more widespread usage.

Ultrasound is widely used in both the antenatal and perinatal imaging for live foetuses and children. It is familiar and understandable to parents and referring clinicians and has also been shown to identify abnormalities in major body organs in the perinatal postmortem setting. Early studies have demonstrated reasonable sensitivity and specificity for whole body diagnoses of approximately 75% and 83.3%, respectively [8], although some abnormalities may be challenging to detect, namely cardiac anomalies with sensitivity rates ranging from 18.2–50% [8–10]. Given the lower cost of ultrasound imaging, and therefore greater affordability and ease of access, it may provide a pragmatic solution to parents who decline conventional invasive autopsy but lack access to other cross-sectional imaging techniques.

Whilst many healthcare professionals in theory possess the technical skills to perform postmortem ultrasound (e.g. sonographers, radiologists, foetal medicine doctors, obstetricians), many are unsure where to begin, what to look for and how to report such studies. This article therefore aims to provide such information to those wishing to start performing perinatal postmortem ultrasound examinations. We provide advice on imaging techniques, views to acquire, technical considerations and some commonly encountered ‘normal postmortem appearances’. The methods described for conducting a thorough ultrasound examination are based upon our own experience of postmortem sonography in a paediatric tertiary referral centre and radiology research unit, with experience of postmortem imaging in a wide range of settings.

## Perinatal deaths

Perinatal death rates and reasons for their occurrences vary considerably across Europe, ranging from 4.6 per 1000 births in Germany to 12.4 per 1000 births in Latvia, with approximately 36,000 annual perinatal deaths across Europe [11–13]. The reasons for these losses can be broadly classified into those relating to maternal health issues (e.g. thrombophilia), placental and cord abnormalities, obstetric complications, acquired (infection) or congenital foetal anomalies [14].

Of these classifications, the commonest indication for postmortem imaging is in assessment of the latter subgroup—namely developmental foetal anomalies and perinatal complications (e.g. intra-cranial haemorrhage). The identification of these pathologies can help account for foetal demise and further define or confirm antenatal imaging findings, particularly following termination of pregnancy. It is also important to bear in mind when counselling parents and clinicians prior to imaging that despite full and thorough investigations, there are still a significant number of perinatal deaths where a cause for foetal demise is not discovered and the cause remains ‘undetermined’ [15, 16].

### Consent and referral indication

Parental consent is vital in all aspects of a perinatal autopsy and, at our institution, permission to perform all postmortem imaging studies are included in our standard autopsy consent forms [17]. Whilst postmortem ultrasound is non-invasive and does not alter the child’s body in any way, we require parental consent for use of any images to be retained and used for research, teaching and educational content.

At present, there are no clear indications or referral criteria for perinatal postmortem ultrasound, as there is limited data to support in which cases it may be of most use. Clearly structural abnormalities such as those identified on antenatal imaging would be most readily identifiable, but confirming the presence or absence of infection may be challenging [7, 8, 13].

### Timing after death

Immediately after receipt of the body to our mortuary, the child is kept in a body bag and in cold storage, at a temperature of 4 °C until imaging can be performed. From personal experience, optimal timing for ultrasonography should be as close as possible to the time of death or delivery; however, there is no evidence of a decline in diagnostic accuracy rates with increasing postmortem interval timing (i.e. time between delivery and imaging). Ultrasound performed for intra-uterine deaths are usually of a lower diagnostic quality in cases where a prolonged intra-uterine retention period has elapsed (due to maceration related changes [18]). These factors are usually unavoidable, given logistical delays in referrals from other hospitals or maternity units. Nevertheless, we still obtain adequate imaging with postmortem intervals of up to 14 days.

### Patient preparation

The body can be scanned immediately following removal from cold storage and does not strictly speaking need to be left to warm at room temperature. Nevertheless, one should be aware that cold storage has been reported to alter tissue properties at CT and MRI imaging [19, 20], and Okuda et al. [21] have postulated that a reduction in the reflection coefficient between fat and soft tissues at colder temperatures could result in diminished ultrasound image contrast. In reality, the changes in image quality between a cold and warm foetus are probably not large enough to present diagnostic challenges, although this has not been comprehensively measured.

Tissue fixation in formalin prior to imaging is also unnecessary in our experience, but may allow for reduced body tissue laxity and is used in some centres for foetuses < 15 weeks gestation in order to preserve intra-cranial anatomy which is prone to maceration [9].

### Scanning environment, equipment and operator

We use standard paediatric postmortem sonographic equipment, but have a dedicated machine for postmortem imaging which is kept in the mortuary. In the absence of a separate machine, reserving certain ultrasound probes for postmortem use may be prudent to minimise cross-contamination and transmission of infection, or else the use of antibacterial wipes and transducer covers will suffice. It is also important to be mindful of adequate privacy in the scanning cubicle, and in some hospitals, this may necessitate postmortem imaging being performed outside of routine working hours.

In general, a high-resolution probe is most effective at visualising the internal body structures for perinatal cases. We recommend tailoring the probe choice to the case, but we have obtained diagnostic image quality using both 2–8 MHz and 7–16 MHz high-frequency linear probes, although other authors have used high-frequency curvilinear probes (such as those used for transvaginal studies due to their smaller footprint), or a lower frequency curvilinear probe for larger subjects [22]. 3D/4D volume acquisitions have been assessed [22], but not widely adopted, and the additional benefit of postmortem 3D ultrasound imaging over 2D imaging has not been thoroughly investigated. Conventional B-mode imaging is therefore suitable and recommended for the entire study. Due to lack of patient movement and cardiovascular output, colour Doppler and M-mode function is not required.

The ultrasound imaging pre-set we use is the same as for imaging superficial musculoskeletal lesions (termed ‘MSK Enhanced’ setting on our system), given the small size of our foetuses and superficial nature of many of the organs. The image depth, time gain control, overall gain and focal zones (both position and number) are amended on a case-by-case basis to acquire the best possible diagnostic quality images. In general, harmonic imaging has not been found to be very useful given the reduced depth and small size of our subjects [23]. Our postmortem studies are performed by a paediatric radiologist although similarly trained sonographers and foetal medicine clinicians may also be suitable.

## Imaging tip

**Fig. 1:**
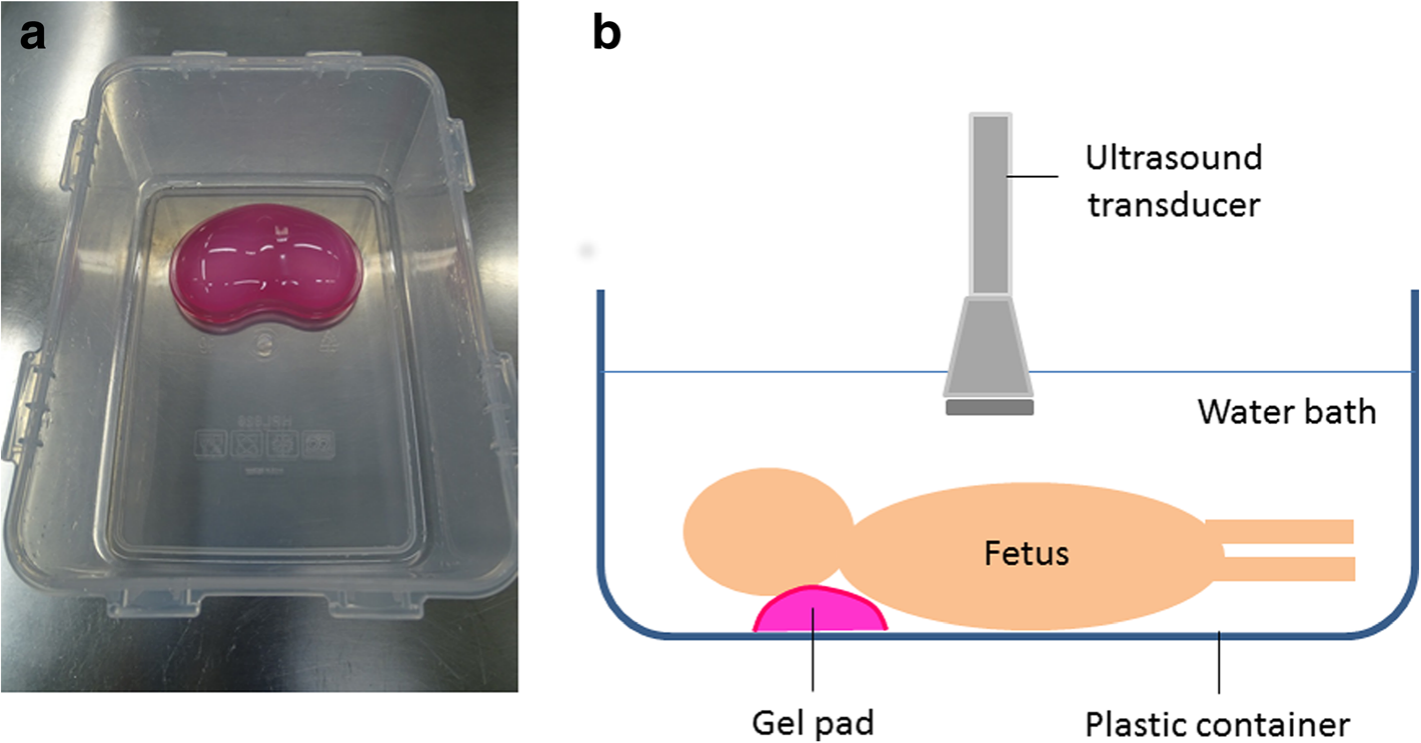
Photograph of an empty 2 L plastic rectangular container (**a**) with a pink silicon gel pad for postmortem ultrasound water bath. Diagram demonstrating the set-up of the water bath (**b**). The container is filled with water, left to rest for 15 min and then the foetus placed in the bath with neck supported by gel pad. The ultrasound transducer is partly submerged in the water

**Fig. 2:**
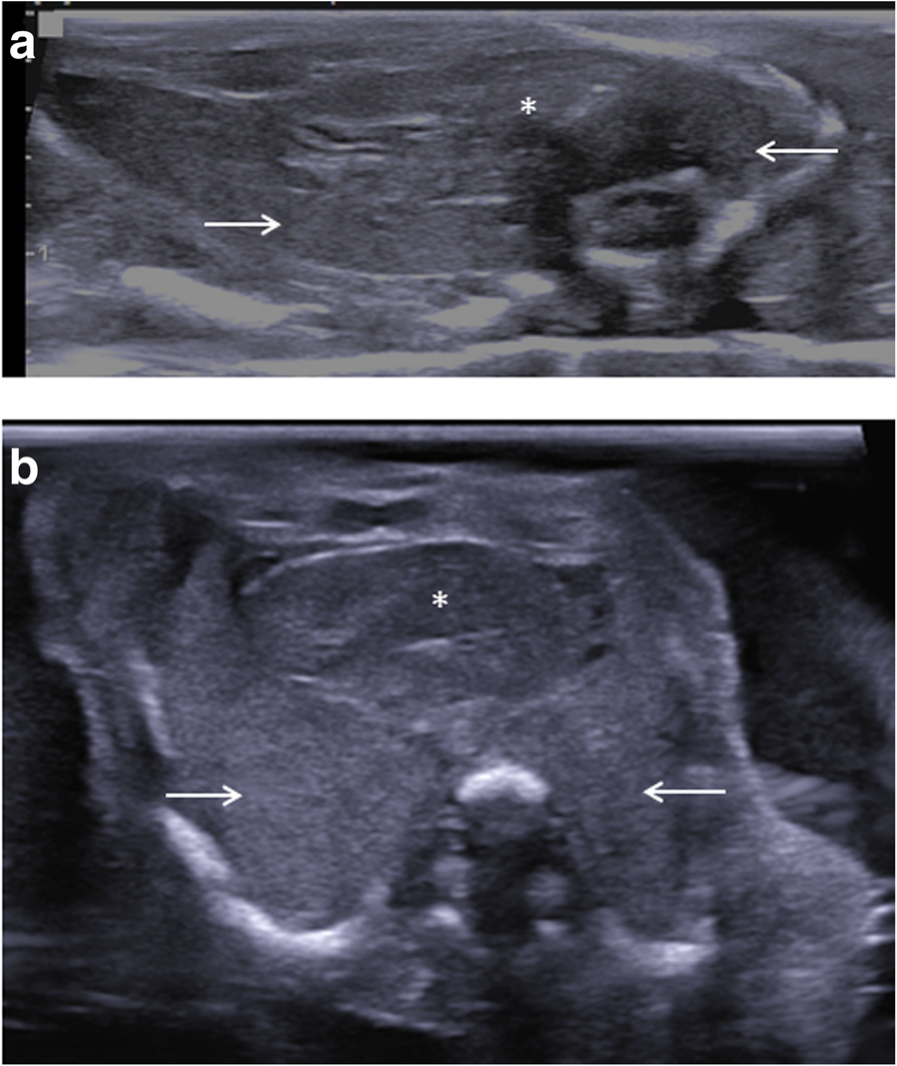
Two transverse postmortem ultrasound images of the chest in the same 23-week gestational-aged foetus, obtained 4 days after death. Postmortem ultrasound performed outside the water bath (**a**) and with foetus in the water bath (**b**). Notice how the thoracic cage was collapsed both due to body laxity and transducer compression in image (**a**), but how there is improved visualisation of the heart (asterisk) and lungs (white arrows) using the water bath (**b**) as the chest expands

Due to the early gestational ages and therefore smaller size of many perinatal cases (compared with live neonatal imaging), image optimisation and visualisation can be an issue, particularly, if there is a lack of complete contact between the footprint of the ultrasound transducer and the foetal body. In order to overcome such a problem, it is possible to use the ‘water bath’ technique (similar to the method used for imaging small inflamed and tender joints in live children [24] Figure 1). This is performed by placing the foetus in a container of cold, still water at a depth just sufficient to submerge the body and head. A gel pad can be used and placed beneath the foetus to allow for stabilisation of the body underwater and prevention of body movement by ripples during scanning. The images obtained may be of slightly reduced spatial resolution; but there is usually much better visualisation of the internal organs owing to improved sonographic wave transmission through the water to greater skin surface area and, since there is no direct pressure of the probe on the body, there is reduced distortion and compression of internal structures (Fig. 2).

An alternative approach to the water bath technique is covering a small foetus in a layer of ultrasound transmission gel (at approximately 0.5 cm depth, as described by Votino et al. [22]). We would not recommend this technique as large quantities of ultrasound gel causes more difficulty in cleaning the body after scanning, and residual gel left on the body can contribute to fungal growth on the skin. For this reason, antiseptic cleaning after scanning is essential, as is ensuring that all gel is removed and the body is dry.

## Imaging and reporting algorithm

Given the lack of aeration of foetal lungs in the majority of perinatal deaths, a whole body ultrasound including imaging of the mediastinum and lungs is possible and visualisation of the majority of the internal body organs can be comprehensively studied. A complete postmortem ultrasound assessment usually takes between 15 and 30 min depending on patient size and complexity.

As with all types of imaging assessment, a consistent and methodical approach should be adopted. We recommend performing and reporting the whole body ultrasound on a ‘systems’ basis, as this is similar in structure to the autopsy reports provided by pathologists. By this method, the body is divided into the following systems: ‘neurological’, ‘cardiac/thoracic’, ‘abdominal’ and ‘musculoskeletal’. We report each system separately with abnormalities pertaining to each area documented. A table listing these systems with recommended ultrasound views, orientation and diagnoses to consider are given in the Additional file 1: Tables S1–S3). We also further discuss these below.

### Brain

**Fig. 3:**
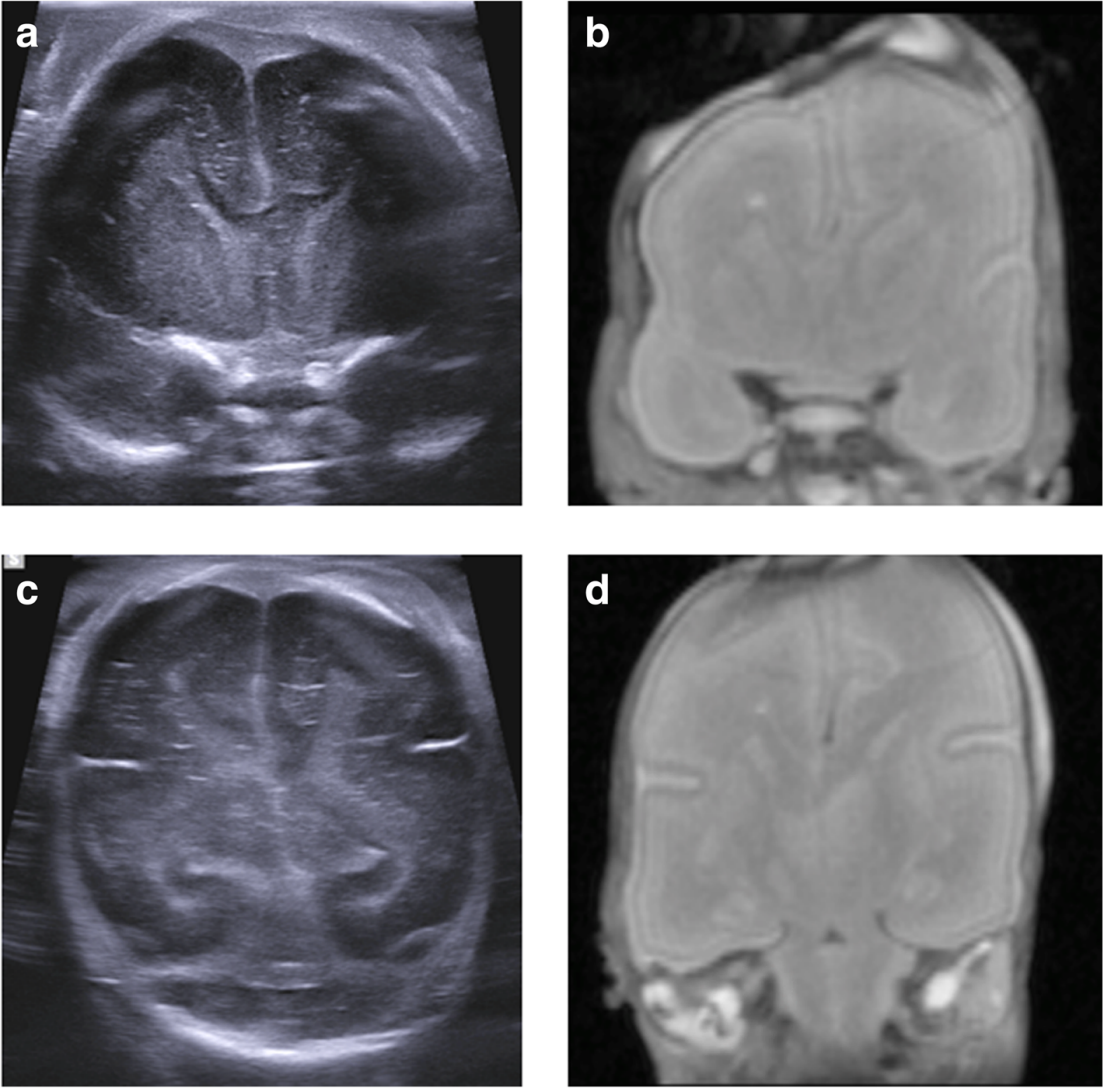
Paired coronal postmortem ultrasound images (obtained in the water bath) and T2-weighted postmortem MRI at 1.5 T in the same 20-week gestational-aged foetus. The images were obtained 4 days after death, demonstrating normal anatomy. Images through the frontal horns (**a**, **b**) and the posterior horns (**c**, **d**) of the lateral ventricles demonstrate how detailed anatomy of the brain, including sulcation and appearances of the ventricles can be adequately reviewed by ultrasound, and not necessarily require further cross-sectional imaging

**Fig. 4:**
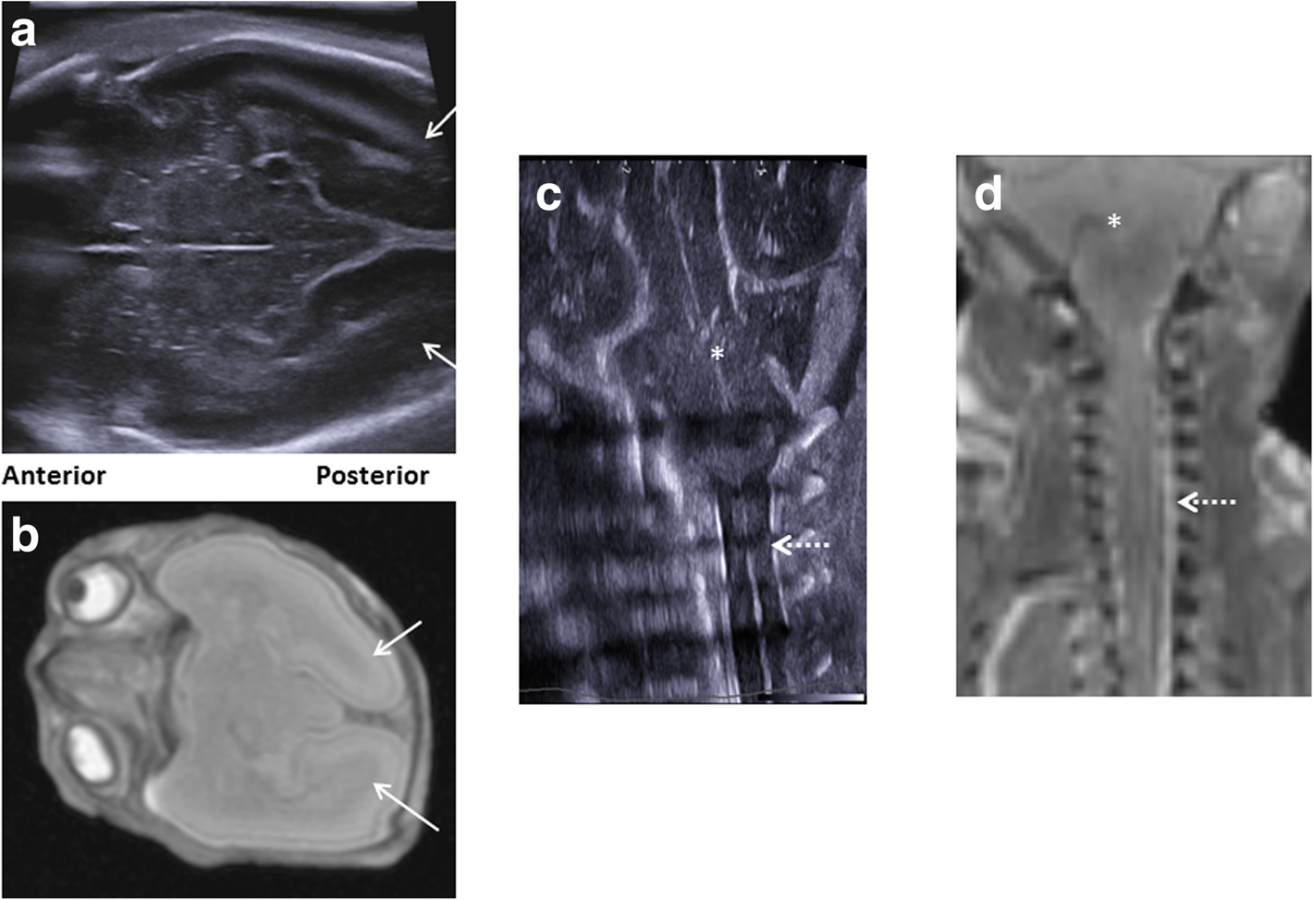
Paired postmortem ultrasound and T2-weighted MRI imaging at 1.5 T are shown in this 20-week gestation foetus, 4 days after death (same case as Fig. 3). The inclusion of additional sonographic views via the left sphenoid fontanelle (**a**) provides clear images of the midbrain and occipital lobes (white arrows), as the comparison with similar view on MRI (**b**) demonstrates. The left trans-mastoid view of the brain at ultrasound (**c**) shows clearly the cerebellum (asterisk) and upper cervical spinal canal (dotted white arrow), comparable in detail as the matched T2-weighted postmortem MRI (**d**)

Coronal and sagittal views can be performed through the anterior fontanelle (Fig. 3), as in live neonatal cranial imaging. Additional views via the sphenoidal fontanelle (to obtain ‘trans-temporal’ views) and via the mastoid fontanelle (for ‘trans-mastoid’ views) provide imaging of the posterior fossa and brain stem including the upper cervical spine (Fig. 4).

**Fig. 5:**
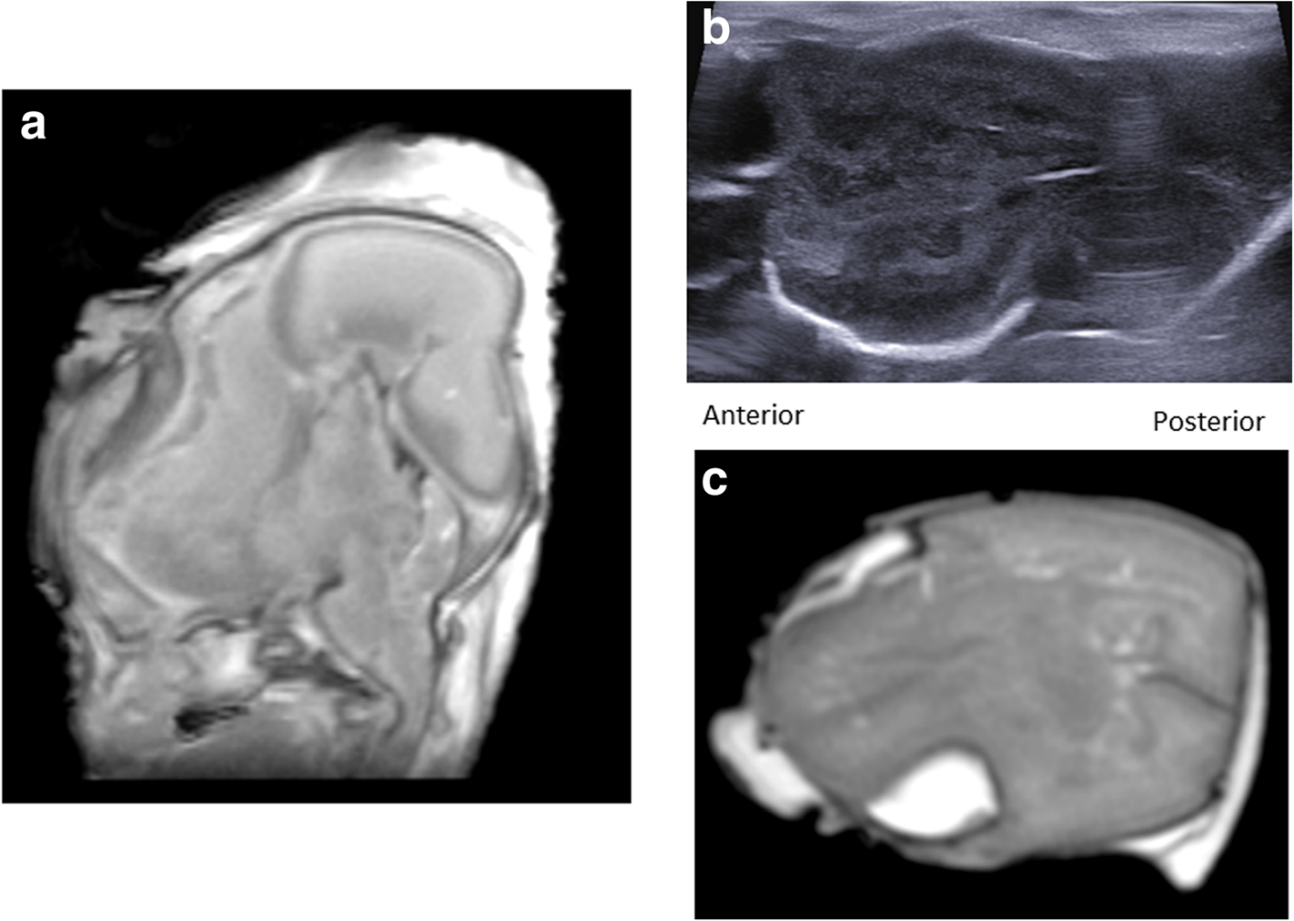
Images of delivery and maceration related changes that degrade postmortem ultrasound image quality in a 22-week gestational-aged foetus. Sagittal T2-weighted postmortem MRI at 1.5 T (**a**) through the midline shows soft tissue oedema of the scalp, overlapping cranial sutures and underlying brain maceration. On the corresponding left trans-temporal view of the brain at postmortem ultrasound (**b**) and at axial postmortem MRI (**c**) at the level of the midbrain, it is difficult to delineate normal brain parenchymal architecture. In such cases, the imaging is described as ‘non-diagnostic’ and useful information cannot be gleaned by either imaging modality

It is important to note that due to the laxity of soft tissues, head moulding and overlapping of cranial sutures in postmortem cases, the quality of images may not always be diagnostic. Intra-cranial gas from postmortem changes and extraction-related deformities can obscure views of the brain (Fig. 5). Typically, the coronal views are the most diagnostically useful (rather than the sagittal views) given distortion of the midline.

When diagnostic quality images are obtained, an assessment can be made on the gestation-appropriate gyration and sulcation of the brain, assessment of midline structures such as corpus callosum (with all components typically visible on antenatal ultrasound imaging by 18 weeks gestation [25]), intra-cranial masses, haemorrhage and ventricular size. With regards to ventricular dilatation, it is worth noting that despite an antenatal history of ventriculomegaly, shifts in cerebrospinal fluid dynamics occurring after death can result in an apparent ‘resolution’ of this appearance and should not be interpreted as a discrepancy or false positive result by prenatal imaging [26].

### Spine

**Fig. 6:**
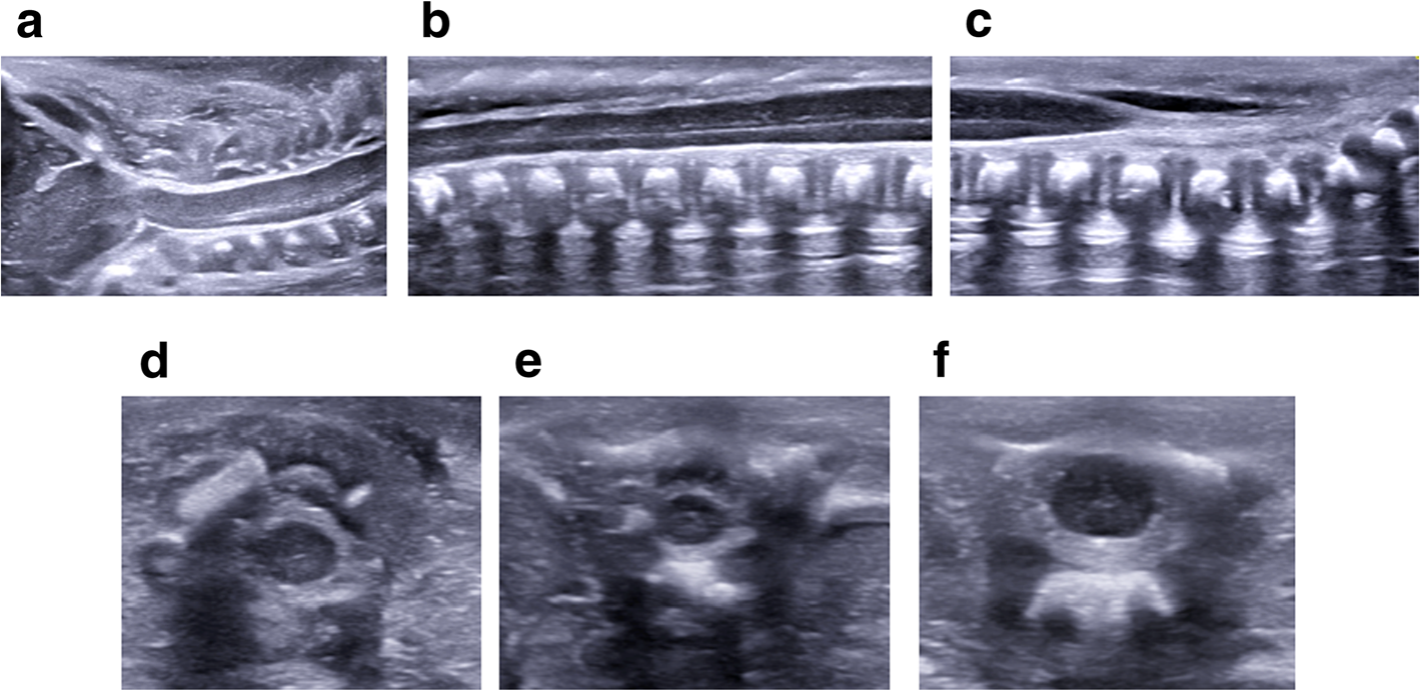
Sagittal postmortem ultrasound images of a normal cervical spine (**a**), thoracolumbar spine (**b**) and lumbosacral spine (**c**) in a 21-week gestational-aged foetus, obtained 12 days after death. Corresponding transverse views of the cervical (**d**), thoracic (**e**) and lumbar (**f**) spinal cord are also shown from a routine normal postmortem ultrasound examination

The views of the spine obtained at postmortem ultrasound are similar to those in live neonatal imaging—namely sagittal views of the conus medullaris, sacrum and transverse images of the cervical, thoracic and lumbar spine (Fig. 6). The pulsatile movements of the filar roots will be absent.

Assessment should be directed at excluding neural tube defects [27] or caudal regression syndrome, identifying vertebral segmental anomalies and spinal dysraphism. Where these are suspected, further characterisation with skeletal radiographs (i.e. babygrams, skeletal survey) can help delineate the pathology better, if not already performed [2].

A useful tip to remember to avoid misdiagnosis of a tethered cord is that gestational age can have a significant impact upon the normal expected level of the conus medullaris. One postmortem series reported that only 50% of foetuses at 26 weeks gestational age have a conus at the L3 vertebral level or above. With earlier gestations, the conus can be normally expected to lie at the L4/5 level or lower [28].

### Thorax

All foetuses who have not breathed (miscarriage, stillbirths or following termination of pregnancy) will still have unaerated, fluid-filled lungs, and some neonates may have made respiratory effort at birth or undergone failed resuscitation and thus present at postmortem with partially aerated lungs. The air within the lungs can obscure adequate sonographic imaging of the lobar anatomy and also clear views of the heart.

**Fig. 7:**
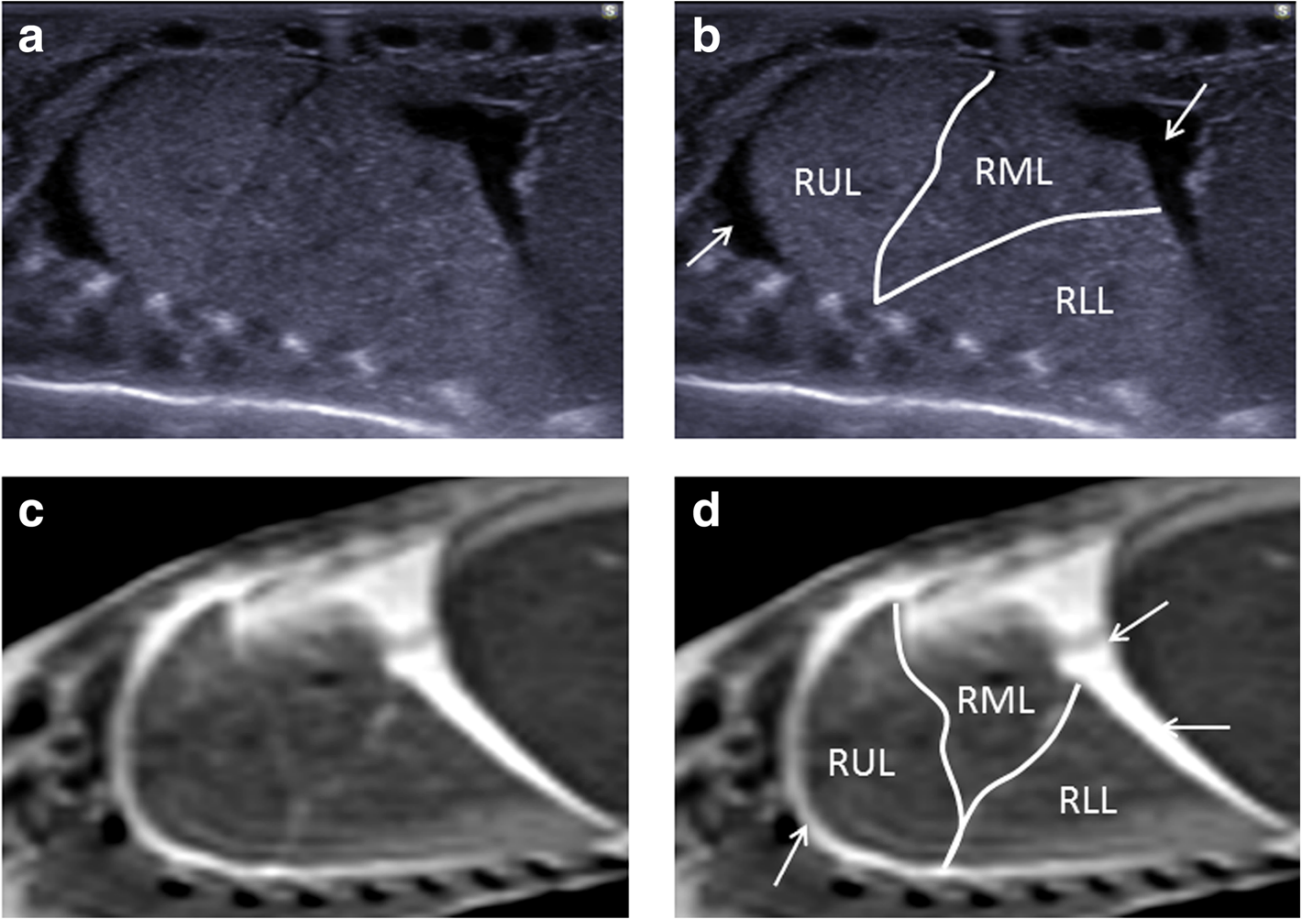
Paired sagittal postmortem ultrasound (**a**, **b**) and T2-weighted postmortem MRI images at 1.5 T (**c**, **d**) of the right lung in the same 18-week gestational-aged foetus, 12 days after death. These images clearly demonstrate the three different pulmonary lobes (RUL—right upper lobe, RML—right middle lobe, RLL—right lower lobe). The solid white arrows on the labelled images show normal expected physiological pleural fluid seen as part of postmortem change and should not be described as pathological

**Fig. 8:**
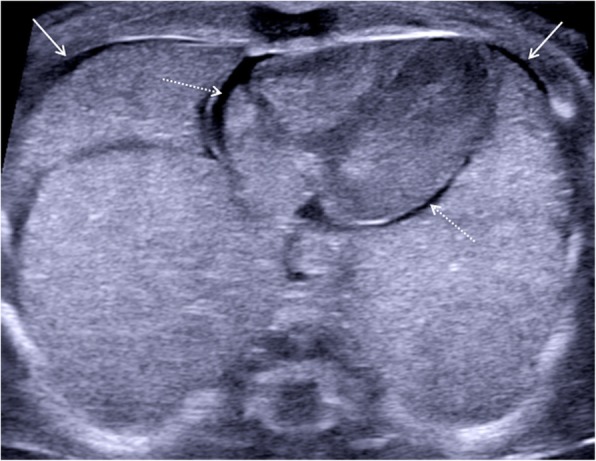
Transverse postmortem ultrasound image through the chest in a 19-week gestational-aged foetus, obtained 8 days after death showing normal postmortem fluid accumulation within the pleural spaces (solid white arrows) and pericardium (dotted arrows) bilaterally. These are commonly seen and described as ‘postmortem changes’ and should not alarm the ultrasound operator

Assessment of the thorax is usually limited to the assessment and symmetry of lobes of each lung (Fig. 7) and presence of pulmonary lesions (e.g. cystic structures which may include bronchopulmonary foregut malformations) or congenital diaphragmatic hernias. Small pericardial and pleural effusions are common and attributable to expected postmortem change [29, 30] (Fig. 8). It is sometimes possible to visualise the fluid-filled trachea, bronchi and also a collapsed oesophagus.

**Fig. 9:**
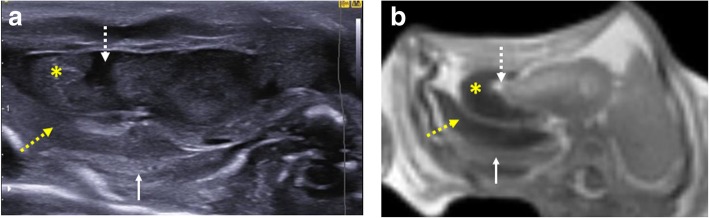
Transverse imaging through the lower thoracic cavity in a 20-week gestational-aged foetus, 2 days after death following termination of pregnancy for suspected renal agenesis. Termination was performed by in utero foetal intra-cardiac injection of potassium chloride. Postmortem ultrasound (**a**) and postmortem T2-weighted MRI at 1.5 T (**b**) both demonstrate haemorrhage within the right pericardial (yellow asterisk) and pleural space (yellow dotted arrow). It is also possible to visualise the ‘normal’ consolidated right lower lobe of the lung (white solid arrow) and a small pericardial effusion (white dotted arrow). These are typical foeticide-related changes and iatrogenic in nature

Termination of pregnancy involving foeticide (i.e. intra-cardiac injection of a toxic agent, such as potassium chloride) can cause haemorrhage (i.e. fluid with internal debris) and gas within the pericardium and pleural spaces, and this should be recognised as iatrogenic and correlated with the antenatal history [31] (Fig. 9).

### Cardiac and vascular imaging

**Fig. 10:**
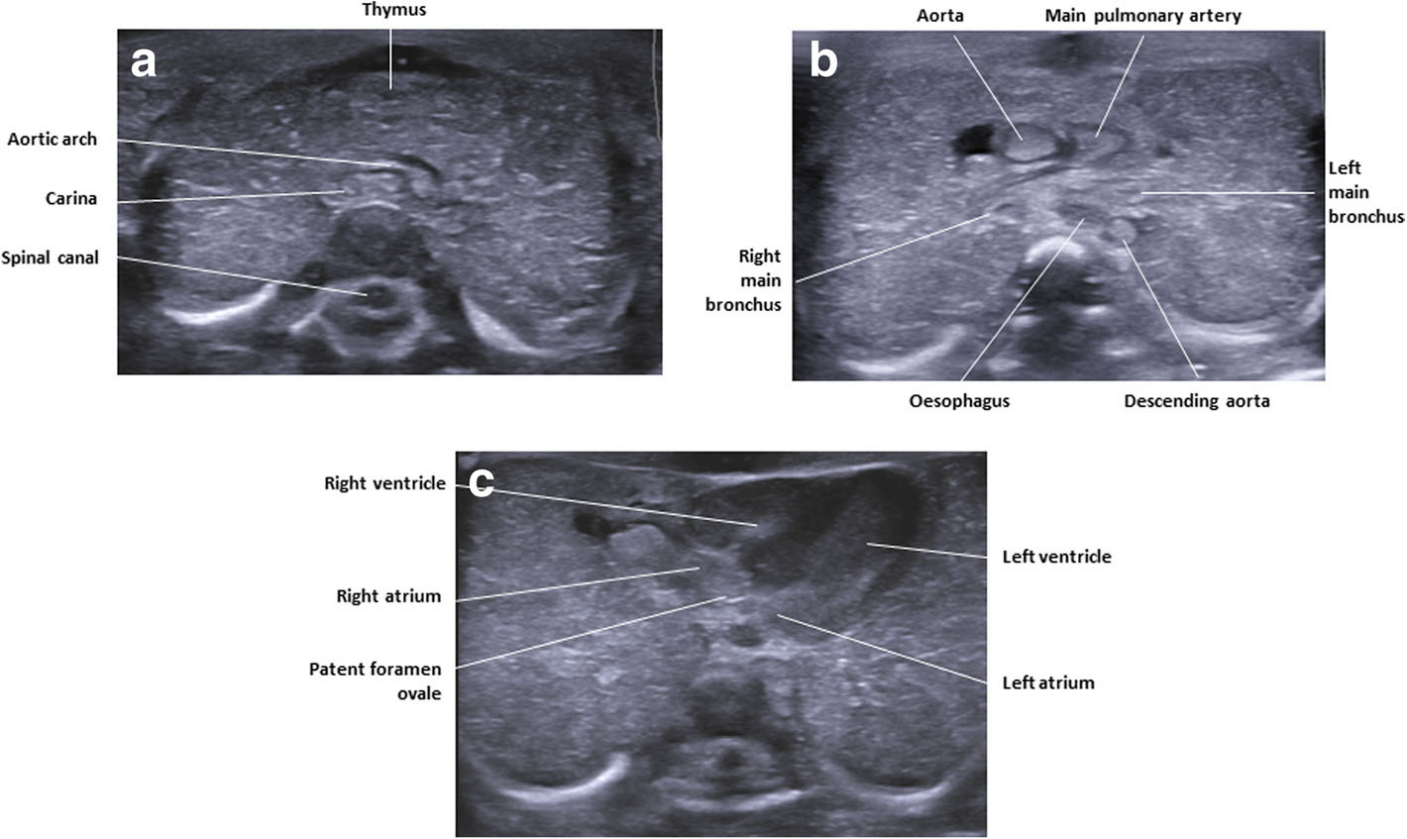
Labelled transverse postmortem ultrasound images of the chest in a 20-week gestational-aged foetus, obtained 4 days after death demonstrating normal anatomy. The labelled images are taken at level of the aortic arch (**a**), level of the main outflow tracts (**b**) and at the biventricular level of the heart (**c**). They serve to demonstrate the detail that can be gained with ultrasound imaging in a well-preserved foetus

Conventional cardiac views can be difficult to obtain at postmortem ultrasound. This may be in part due to body distortion and the small size of the heart at the limits of ultrasound resolution at earlier gestation. Lack of cardiovascular output and therefore colour Doppler flow also hamper structural assessment of the heart, which may account for the lower diagnostic accuracy rates for this body area, as alluded to in the introduction. Nevertheless, by obtaining transverse and sagittal views through the heart, it is still possible to glean useful information regarding ventricular wall thickness, the orientation of the outflow tracts (Fig. 10) and presence of septal defects.

**Fig. 11:**
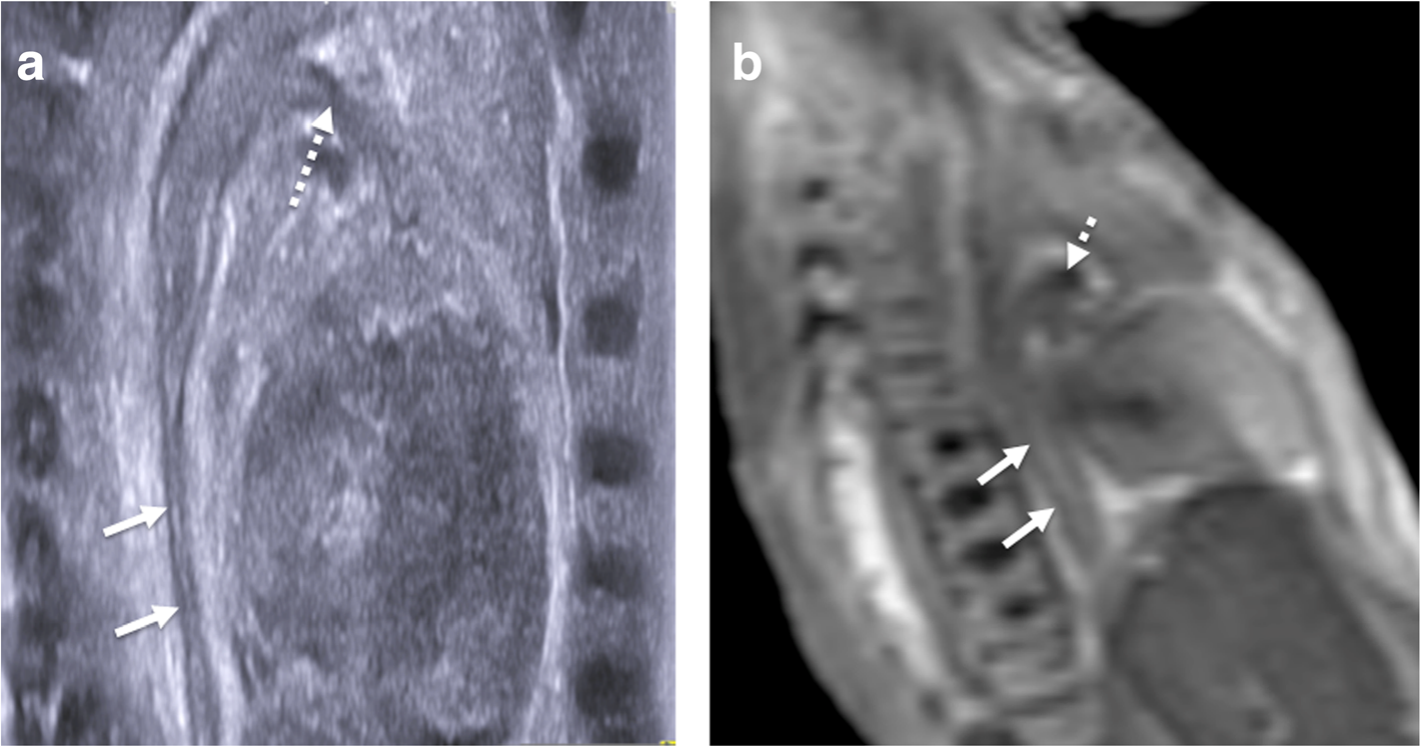
Paired sagittal midline imaging of the chest, in a 20-week gestational-aged foetus obtained approximately 1 week after death. The postmortem ultrasound imaging (**a**) and T2-weighted MRI imaging at 1.5 T (**b**) both clearly demonstrate the usual appearances of haemostasis and collapse of the descending aorta (solid white arrows) and also prominent patent ductus arteriosus (dotted arrow)

Where there is a large intra-thoracic mass (e.g. congenital diaphragmatic hernia), it is also possible to make a comment regarding the displacement of the mediastinum. Imaging of the aortic arch, superior vena cava and pulmonary veins are sometimes possible (Fig. 11), but usually collapsed.

### Abdomen

**Fig. 12:**
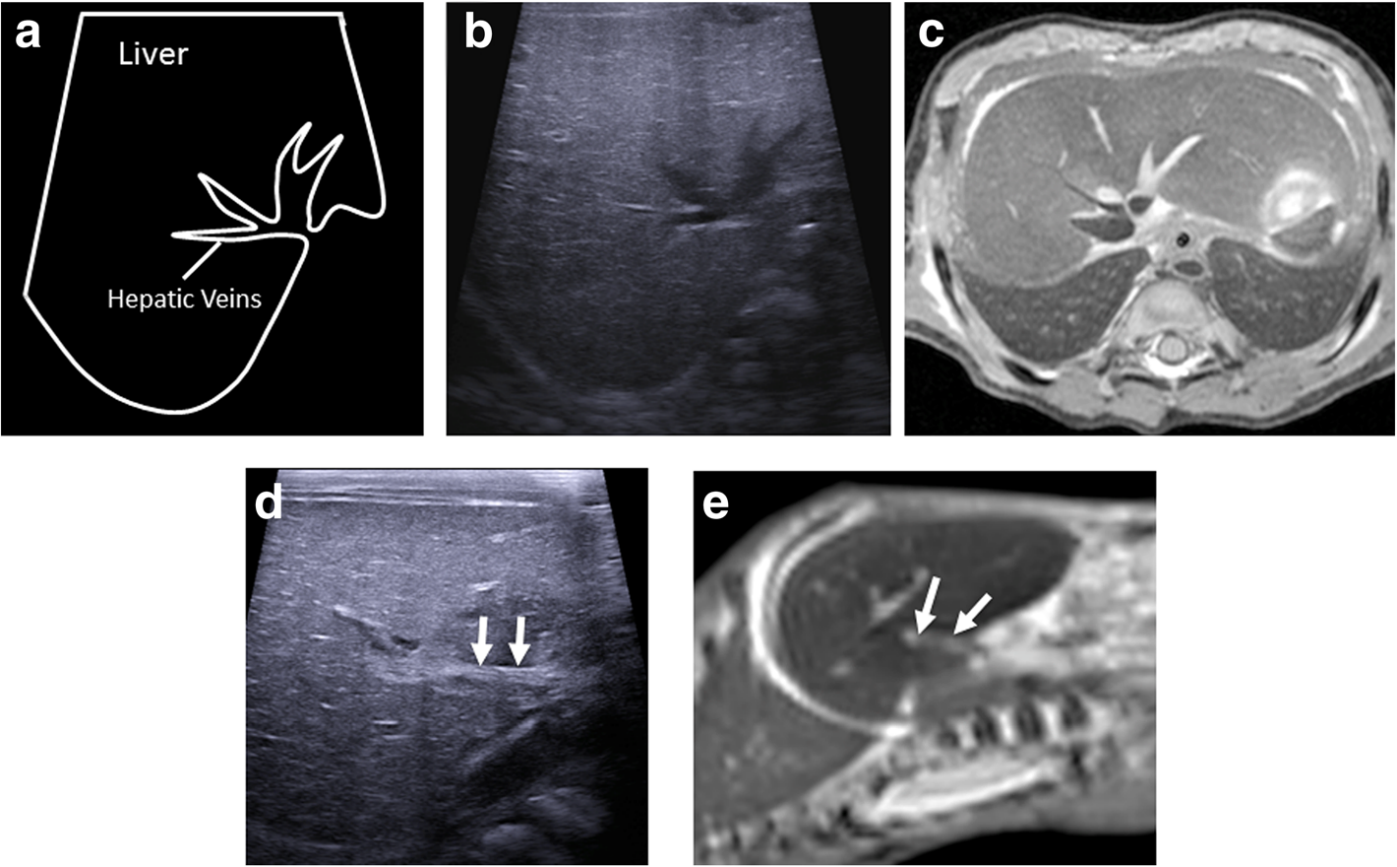
Matching transverse upper abdominal views in labelled diagram format (**a**), postmortem ultrasound (**b**) and T2-weighted MRI at 1.5 T imaging (**c**) of the same 36-week gestational-aged foetus 3 days after death demonstrating haemostasis in the hepatic veins. The corresponding sagittal postmortem ultrasound (**d**) and matched T2-weighted MRI (**e**) views of the thrombosed main portal vein (white arrows) are also shown. This imaging example shows how even with decent sonographic windows in a well-preserved foetus, the common bile duct is quite difficult to delineate

**Fig. 13:**
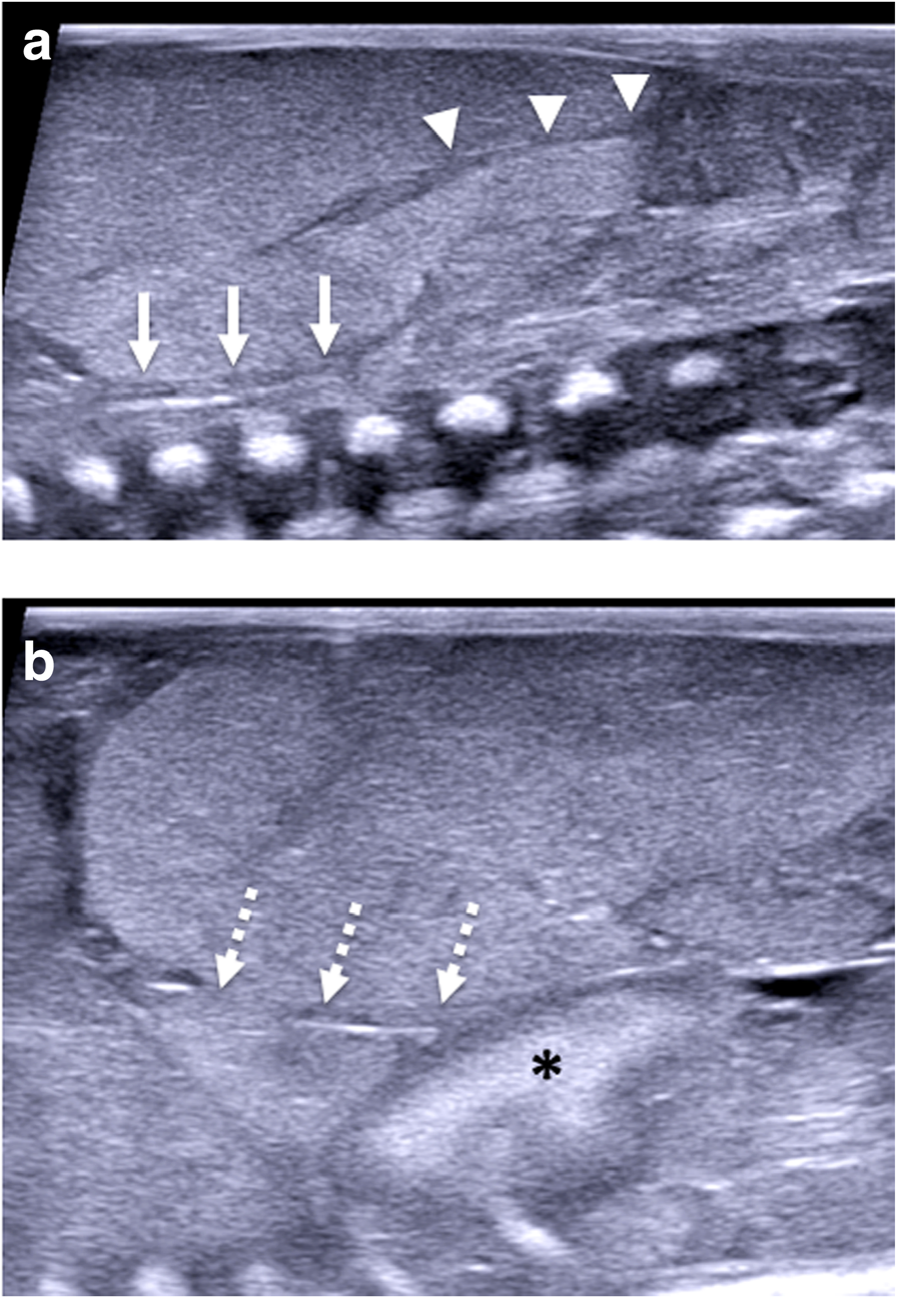
Sagittal postmortem ultrasound images of the upper abdomen in a 19-week gestational-aged foetus, obtained 8 days after death. Images through the abdominal aorta (solid arrows) (**a**) and ductus venosus (arrow heads) are both collapsed. The imaging through the inferior vena cava (dotted arrows) (**b**) show similar appearances. Another finding of note is the echogenic, prominent appearing right adrenal gland (asterisk). This is the usual expected appearance of the adrenal gland on perinatal postmortem ultrasound examinations

All intra-abdominal organs are methodologically assessed in both the transverse and sagittal planes, as in live cases. The hepatic, renal and intra-abdominal vessels are commonly collapsed and therefore their assessment is difficult (Figs. 12 and 13). The common bile duct is also nearly impossible to identify; however, the presence or absence of the gallbladder is usually possible.

**Fig. 14:**
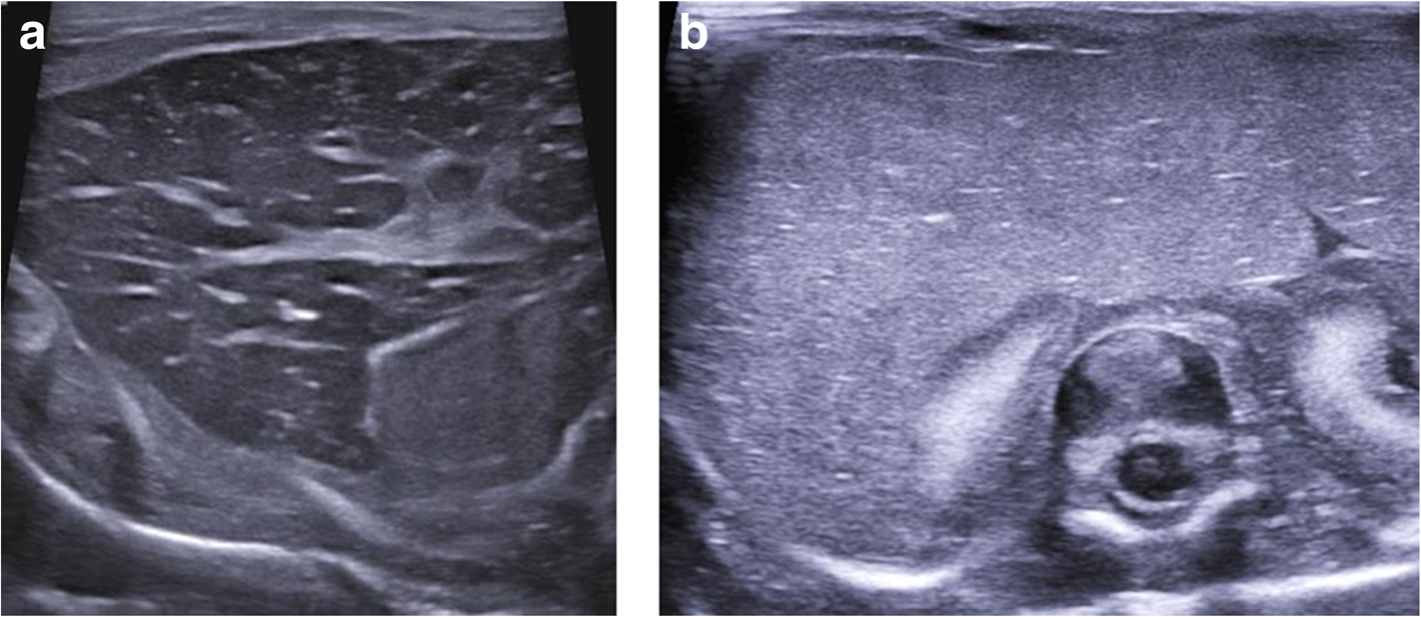
Transverse postmortem ultrasound images of a normal liver in two different foetuses, both acquired 12 days after death. The liver in image (**a**) demonstrates marked periportal echogenicity and was obtained in a 32-week gestational-aged stillborn foetus. In image (**b**), there are fewer periportal echoes in this 21-week gestational-aged foetus following termination of pregnancy for oligohydramnios. The causes for these differences is unknown and could relate to different phases of decomposition or gestational age, as both livers were histologically normal at autopsy

**Fig. 15:**
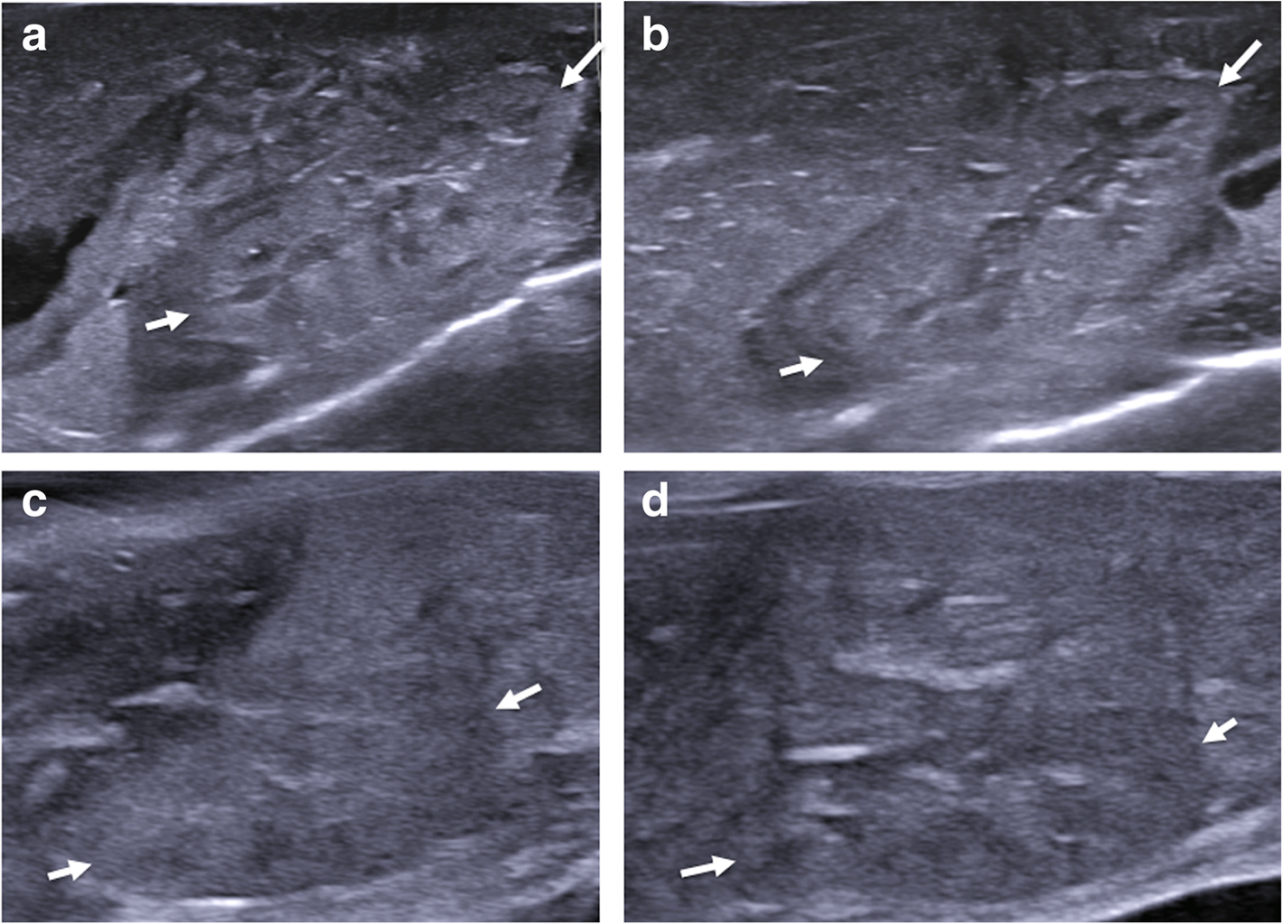
Normal sagittal appearances of the kidneys at postmortem ultrasound in two different foetuses (left column (**a**, **c**)—left kidney, right column (**b**, **d**)—right kidney). The solid white arrows in each of the images demonstrate the upper and lower poles of the kidneys. Images (**a**, **b**) were obtained in a 20-week gestational-aged foetus, 4 days after death. They show the normal expected corticomedullary differentiation. Images (**c**, **d**) were obtained from a stillborn 25-week gestational-aged foetus, 10 days after death. These kidneys lack corticomedullary differentiation and are harder to identify when seen against the background of other solid abdominal viscera and bowel. All kidneys were histologically unremarkable both on antenatal ultrasound imaging and autopsy. The lack of corticomedullary differentiation is likely to relate to autolysis or maceration-related changes, rather than pathological causes

Findings that may be pathological in live neonates, but which are frequently physiological in the postmortem setting, include a small amount of simple ascites, periportal echogenicity in the liver (Fig. 14) and some loss of corticomedullary differentiation of the kidneys (especially with prolonged intra-uterine retention) (Fig. 15).

Septated fluid in the abdomen and large-volume ascites causing abdominal distension is however abnormal and, where present, should raise concern for hydrops or underlying sepsis. In addition, kidneys that appear enlarged, markedly echogenic or have internal cystic structures and pelvicalyceal dilatation are suggestive of underlying renal anomalies. Where kidneys are not identified in the retroperitoneum, a detailed examination of the pelvis should be performed to exclude an ectopic kidney.

**Fig. 16:**
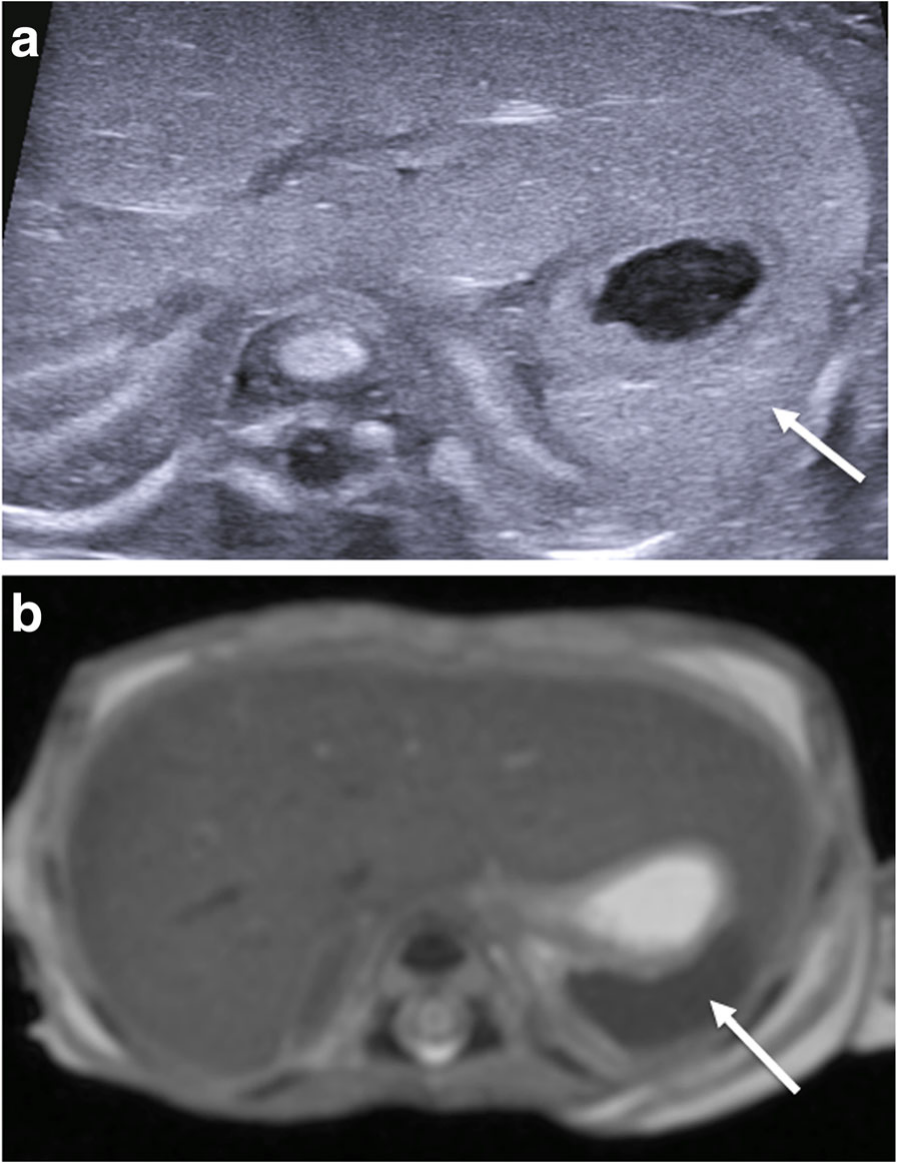
Normal appearances of the liver and spleen in a 19-week gestational-aged foetus following miscarriage, imaged 6 days after death. The transverse postmortem ultrasound imaging (**a**) through the upper abdomen demonstrates a very similar echogenicity of the liver with the spleen (white arrow). The differences between the two organs is much better seen at T2-weighted postmortem MRI (**b**), where both organs demonstrate different signal intensities

**Fig. 17:**
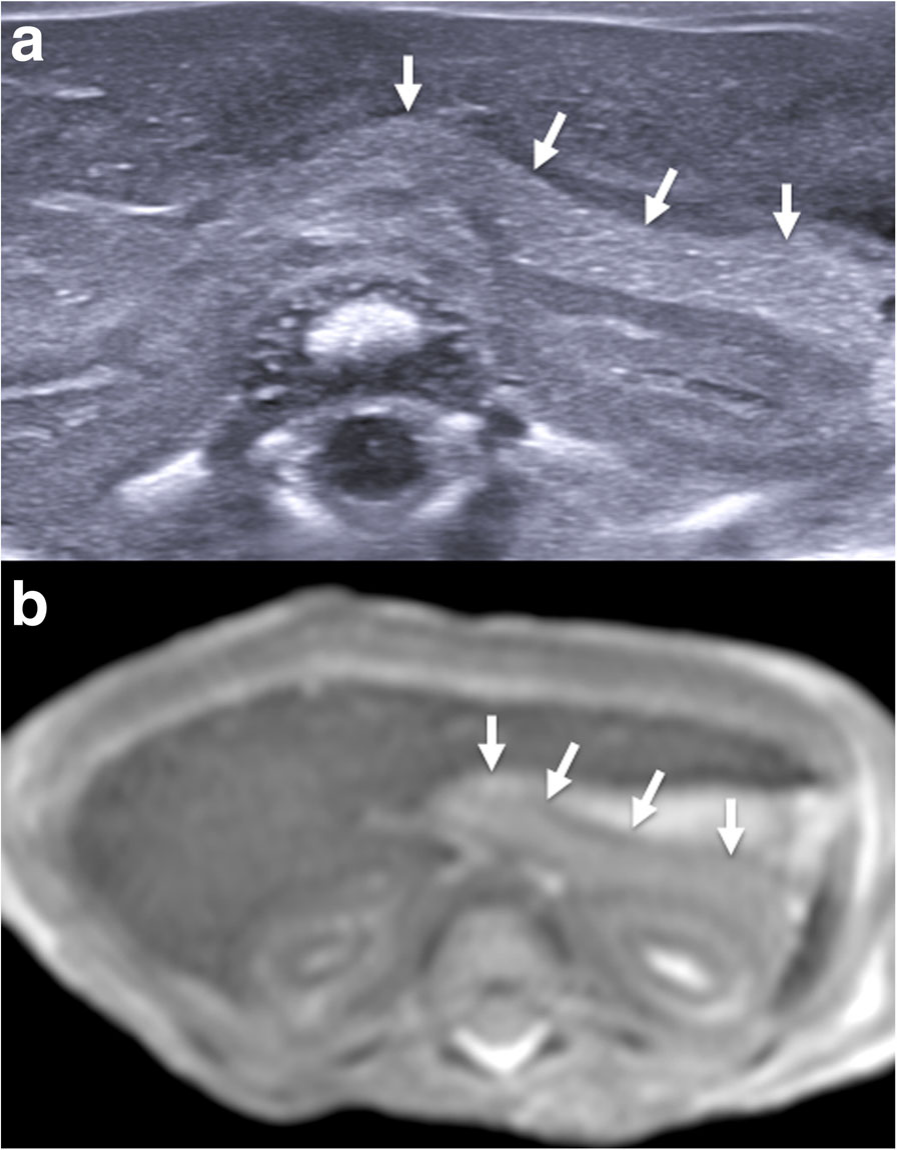
Normal appearances of the pancreas (white arrows) on transverse postmortem ultrasound (**a**) in a 20-week gestational-aged foetus, 4 days after death with corresponding postmortem T2-weighted MRI at 1.5 T (**b**) obtained 12 days after death

One common difficulty in perinatal postmortem ultrasound imaging is in the identification and location of the spleen, particularly in earlier gestation foetuses. The reasons for this are twofold—the first being that the liver can be relatively quite large and the spleen relatively small in size [32] during foetal life and, secondly, the echotexture of the hepatic parenchyma can be very similar to that of the spleen (Fig. 16). Nevertheless, an enlarged spleen can often be identified, which could signify underlying infection or an inborn error of metabolism (e.g. perinatal Gaucher’s [33] and other alloimmune diseases [34]). The pancreas is also occasionally a difficult organ to identify given that it can autolyse early on in the maceration process and appear absent. When present in a non-macerated foetus, the appearances are very similar to ante-mortem ultrasound images (Fig. 17).

**Fig. 18:**
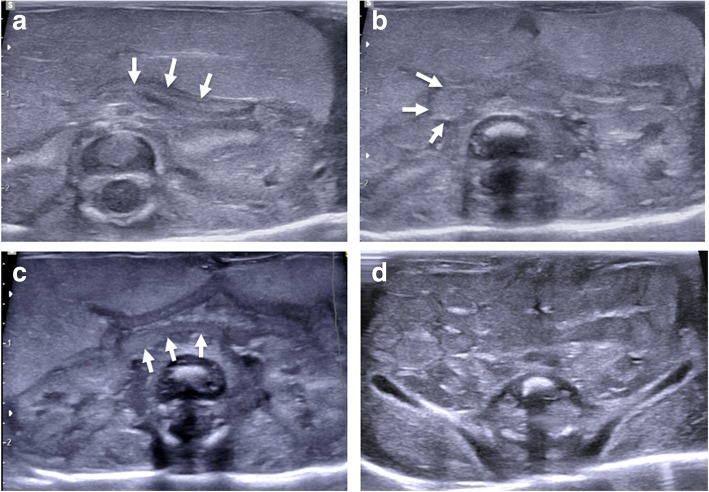
Normal transverse postmortem ultrasound appearances of the bowel in a 19-week gestational-aged foetus, obtained 8 days after death. The first three images (**a**–**c**) demonstrate normal rotation of the bowel. In image (**a**), the gastric pylorus becomes the first part of the duodenum (solid white arrows) and is seen to cross the midline to the right of the abdomen. In image (**b**), the second part of the duodenum is shown (white arrows), and finally, in image (**c**), this becomes the third part of the duodenum as it crosses back to the left side of the abdomen (white arrows). In image (**d**), normal small and large bowel loops in the lower abdomen are seen and meconium filled, without any significant bowel wall thickening or interloop separation

Finally, after all solid viscera are identified and assessed, the bowel and pelvis are examined. Assuming that the anterior abdominal wall is intact and there is no diaphragmatic hernia, commenting upon normal bowel rotation is possible. In this instance, the lack of intraluminal gas is helpful as it allows one to trace the path of the duodenum and assess where it crosses the midline within the retroperitoneum (Fig. 18). This is easier than identifying the orientation of the superior mesenteric artery and vein on postmortem ultrasound which are commonly thrombosed and small in size.

**Fig. 19:**
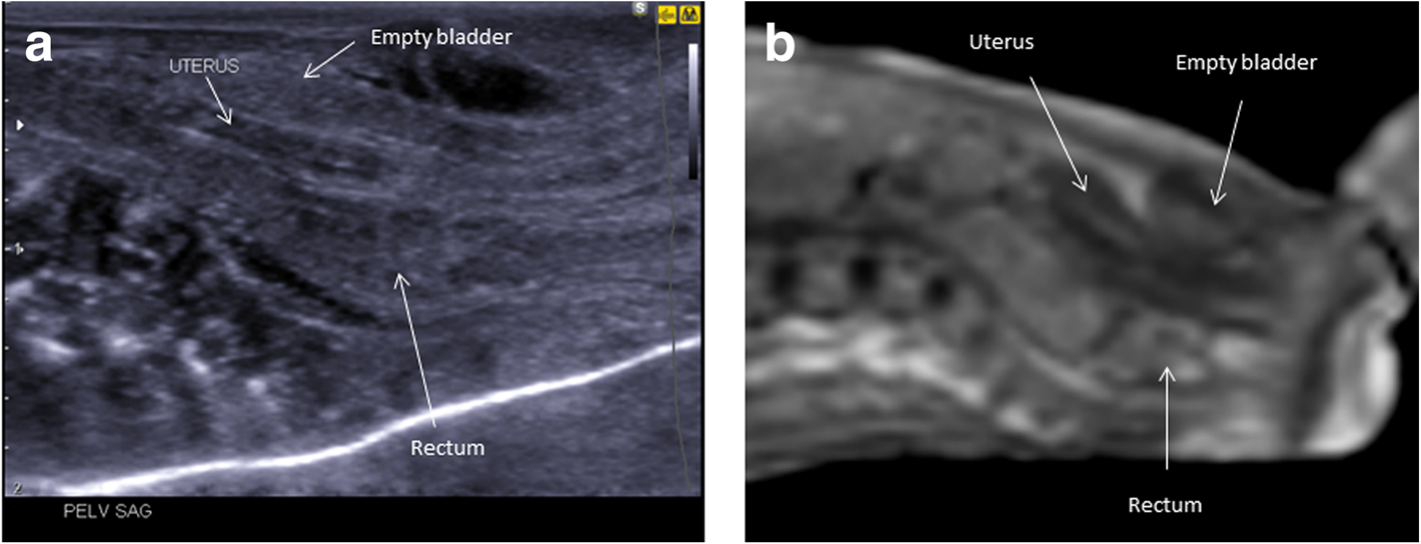
These images demonstrate normal appearances of the pelvic structures in a female 18-week gestational-aged foetus. Sagittal postmortem ultrasound of the pelvis (**a**) and corresponding postmortem T2-weighted MRI at 1.5 T (**b**) have been obtained 12 days after death. The tubular appearances of a normal pre-pubertal uterus, rectum and urinary bladder are all well seen and labelled in these images. Using a high-frequency linear probe, the pelvic anatomy can be better demonstrated on ultrasound rather than MRI

The pelvic structures are best visualised on a sagittal view of the lower abdomen (Fig. 19). It is possible to appreciate the rectum, urinary bladder and uterus (if female). Ovaries are not usually visualised in early gestational aged foetuses, and the testes (in males) can frequently be beyond the limits of image resolution for adequate assessment.

### Musculoskeletal

Postmortem ultrasound assessment for the musculoskeletal system is best reserved for assessment of soft tissue masses, particularly of the head and neck such as venolymphatic (and other vascular) malformations or teratomas [35].

Where further skeletal imaging is needed, radiography or CT for ossified structures can be more helpful. In the perinatal setting, a skeletal survey alone is sufficient to diagnose inheritable bone disorders (i.e. skeletal dysplasias). Whilst several studies have demonstrated the relative futility in performing radiography routinely for all perinatal deaths [2, 36, 37], it still remains common practice in most centres and therefore can be easily referenced if there is any doubt regarding bony appearances during sonographic imaging.

## Conclusions

Perinatal postmortem ultrasound is an easily accessible and simple imaging tool that can allow for visualisation of internal organs and aid the perinatal autopsy. This article has highlighted the key aspects for approaching a postmortem ultrasound examination including patient preparation, imaging protocol and organ assessment. It serves as a guideline for radiologists, sonographers, foetal medicine clinicians and potentially other allied health professionals who have ultrasound skills and are keen to provide a non-invasive imaging autopsy service.

In early cohort studies, postmortem ultrasound has shown high concordance rates with autopsy although certain anomalies, such as thoracic and cardiac pathologies may still pose a challenge and remain a major limitation.

The future of postmortem ultrasound may include usage as a first line ‘screening’ technique to determine which patients could benefit from further postmortem cross-sectional imaging (e.g. PMMR), or for assistance during image-guided organ biopsies in a ‘minimally invasive autopsy’, where tissue samples for genetic or histopathological analysis is required. Both of these topics represent areas of further research on this subject.

## Additional file

Additional file 1: **Table S1.** Imaging protocol for perinatal postmortem ultrasound study—neurological system. **Table S2.** Imaging protocol for perinatal postmortem ultrasound study—cardiothoracic system. **Table S3.** Imaging protocol for perinatal postmortem ultrasound study—abdominal system. (DOCX 24 kb)

## Abbreviations

CT: Computed tomography
MRI: Magnetic resonance imaging
PACS: Picture archiving and communications system
PMCT: Postmortem computed tomography
PMMR: Postmortem magnetic resonance imaging
PMUS: Postmortem ultrasound

## References

[1] Lewis C, Hill M, Arthurs OJ, Hutchinson C, Chitty LS, Sebire NJ (2018). Factors affecting uptake of postmortem examination in the prenatal, perinatal and paediatric setting. BJOG.

[2] Arthurs OJ, Calder AD, Kiho L, Taylor AM, Sebire NJ (2014). Routine perinatal and paediatric post-mortem radiography: detection rates and implications for practice. Pediatr Radiol.

[3] Sieswerda-Hoogendoorn T, Soerdjbalie-Maikoe V, de Bakker H, van Rijn RR (2014). Postmortem CT compared to autopsy in children; concordance in a forensic setting. Int J Legal Med.

[4] van Rijn RR, Beek EJ, van de Putte EM et al (2017) The value of postmortem computed tomography in paediatric natural cause of death: a Dutch observational study. Pediatr Radiol 47:1514–1522 10.1007/s00247-017-3911-0 PMC5608837 28681231

[5] Thayyil S, Sebire NJ, Chitty LS et al (2013) Post-mortem MRI versus conventional autopsy in fetuses and children: a prospective validation study. Lancet 382:223–233 10.1016/S0140-6736(13)60134-8 23683720

[6] Ashwin C, Hutchinson JC, Kang X et al (2017) Learning effect on perinatal post-mortem magnetic resonance imaging reporting: single reporter diagnostic accuracy of 200 cases. Prenat Diagn 37:566–574 10.1002/pd.5043 28342279

[7] Arthurs OJ, Guy A, Thayyil S (2016). Comparison of diagnostic performance for perinatal and paediatric post-mortem imaging: CT versus MRI. Eur Radiol.

[8] Kang X, Shelmerdine SC, Hurtado I (2019). Postmortem examination of human fetuses: a comparison of 2-dimensional ultrasound with invasive autopsy. Ultrasound Obstet Gynecol.

[9] Tuchtan L, Lesieur E, Bartoli C et al (2016) Comparison of postmortem ultrasound and autopsy in 75 cases of perinatal death. Ultrasound Obstet Gynecol 48:156

[10] Prodhomme O, Baud C, Saguintaah M (2015). Comparison of postmortem ultrasound and X-ray with autopsy in foetal death: retrospective study of 169 cases. J Forensic Radiol Imaging.

[11] Arthurs OJ, van Rijn RR, Taylor AM, Sebire NJ (2015). Paediatric and perinatal postmortem imaging: the need for a subspecialty approach. Pediatr Radiol.

[12] de Jonge A, Baron R, Westerneng M, Twisk J, Hutton EK (2013). Perinatal mortality rate in the Netherlands compared to other European countries: a secondary analysis of Euro-PERISTAT data. Midwifery.

[13] Office for National Statistics EaW (2016) Births in England and Wales: 2016: Live births, stillbirths, and the intensity of childbearing measured by the total fertility rate. Available via https://backup.ons.gov.uk/wpcontent/uploads/sites/3/2017/07/Births-in-England-and-Wales-2016.pdf

[14] Nijkamp JW, Sebire NJ, Bouman K, Korteweg FJ, Erwich JJHM, Gordijn SJ (2017). Perinatal death investigations: what is current practice?. Semin foetal Neonatal Med.

[15] Man J, Hutchinson JC, Heazell AE, Ashworth M, Levine S, Sebire NJ (2016). Stillbirth and intrauterine foetal death: factors affecting determination of cause of death at autopsy. Ultrasound Obstet Gynecol.

[16] Basu MN, Johnsen IBG, Wehberg S, Sørensen RG, Barington T, Nørgård BM (2018). Causes of death among full term stillbirths and early neonatal deaths in the Region of Southern Denmark. J Perinat Med.

[17] Judge-Kronis L, Hutchinson JC, Sebire NJ, Arthurs OJ (2016). Consent for paediatric and perinatal postmortem investigations: implications of less invasive autopsy. J Forensic Radiol Imaging.

[18] Montaldo P, Addison S, Oliveira V (2016). Quantification of maceration changes using post mortem MRI in fetuses. BMC Med Imaging.

[19] Sugimoto M, Hyodoh H, Rokukawa M (2016). Freezing effect on brain density in postmortem CT. Legal Med (Tokyo).

[20] Zech WD, Schwendener N, Persson A, Warntjes MJ, Jackowski C (2015). Temperature dependence of postmortem MR quantification for soft tissue discrimination. Eur Radiol.

[21] Okuda T, Shiotani S, Kobayashi T (2016). Principles of foetal postmortem ultrasound: a personal review. J Forensic Radiol Imaging.

[22] Votino C, Cos Sanchez T, Bessieres B (2018). Minimally invasive foetal autopsy using ultrasound: a feasibility study. Ultrasound Obstet Gynecol.

[23] Anvari A, Forsberg F, Samir AE (2015). A primer on the physical principles of tissue harmonic imaging. Radiographics.

[24] Blaivas M, Lyon M, Brannam L, Duggal S, Sierzenski P (2004). Water bath evaluation technique for emergency ultrasound of painful superficial structures. Am J Emerg Med.

[25] Goldstein A, Mesfin FB (2018). Neuroanatomy, Corpus Callosum.

[26] Sebire NJ, Miller S, Jacques TS (2013). Post-mortem apparent resolution of foetal ventriculomegaly: evidence from magnetic resonance imaging. Prenat Diagn.

[27] Shelmerdine SC, Hutchinson JC, Sebire NJ, Jacques TS, Arthurs OJ (2017). Post-mortem magnetic resonance (PMMR) imaging of the brain in fetuses and children with histopathological correlation. Clin Radiol.

[28] Arthurs OJ, Thayyil S, Wade A, Chong WK, Sebire NJ, Taylor AM, Magnetic Resonance Imaging Autopsy Study Collaborative G (2013). Normal ascent of the conus medullaris: a post-mortem foetal MRI study. J Matern foetal Neonatal Med.

[29] Arthurs OJ, Barber JL, Taylor AM, Sebire NJ (2015). Normal perinatal and paediatric postmortem magnetic resonance imaging appearances. Pediatr Radiol.

[30] Barber JL, Hutchinson JC, Sebire NJ, Arthurs OJ (2016). Pleural fluid accumulation detectable on paediatric post-mortem imaging: a possible marker of interval since death. Int J Legal Med.

[31] Shelmerdine SC, Hickson M, Sebire NJ, Arthurs OJ (2018). Post-mortem magnetic resonance imaging appearances of foeticide in perinatal deaths. foetal Diagn Ther.

[32] Ungör B, Malas MA, Sulak O, Albay S (2007). Development of spleen during the foetal period. Surg Radiol Anat.

[33] BenHamida E, Ayadi I, Ouertani I (2015). Perinatal-lethal Gaucher disease presenting as hydrops foetalis. Pan Afr Med J.

[34] Sciard C, Collardeau-Frachon S, Atallah A (2018). Prenatal imaging features suggestive of liver gestational allo immune disease. J Gynecol Obstet Hum Reprod.

[35] Papadopoulou I, Sebire NJ, Shelmerdine SC, Bower S, Arthurs OJ (2015). Postmortem image-guided biopsy for less-invasive diagnosis of congenital intracranial teratoma. Ultrasound Obstet Gynecol.

[36] Kamphuis-van Ulzen K, Koopmanschap DH, Marcelis CL, van Vugt JM, Klein WM (2016). When is a post-mortem skeletal survey of the fetus indicated, and when not?. J Matern foetal Neonatal Med.

[37] Olsen E ØE, Espeland A, Maartmann-Moe H, Lachman RS, Rosendahl K (2003) Diagnostic value of radiography in cases of perinatal death: a population based study. Arch Dis Child foetal Neonatal Ed 88:F521–F524 10.1136/fn.88.6.F521 PMC1763230 14602703

