# Supplementary material for: Perinatal post mortem ultrasound (PMUS): a practical approach
Source: Insights Imaging. 2019 Mar 18;10:35. doi: 10.1186/s13244-019-0723-9 (PMC6423182; doi:10.1186/s13244-019-0723-9)
Supplement: Supplementary file 1 — Table S1. Imaging protocol for perinatal postmortem ultrasound study—neurological system. Table S2. Imaging protocol for perinatal postmortem ultrasound study—cardiothoracic system. Table S3. Imaging protocol for perinatal postmortem ultrasound study—abdominal system. (DOCX 24 kb) [file 13244_2019_723_MOESM1_ESM.docx]

**ADDITIONAL FILES**

**Table S1: Imaging Protocol for Perinatal Postmortem Ultrasound Study – Neurological System**

| **Body System:** | **Body Part:** | **Orientation:** | **Images Obtained & Assessed:** |
| --- | --- | --- | --- |
| **Neurological** | **Cerebral hemispheres** | Coronal sections, probe placed at the anterior fontanelle | - Level of frontal lobes - Level of frontal horns of lateral ventricles - Level of foramen of Monroe and 3rd ventricle - Level of the bodies of lateral ventricles - Level of trigone of lateral ventricles - Level of parieto-occipital lobes |
|  |  | Sagittal planes, probe placed at the anterior fontanelle | - Right and left temporal cerebral lobes - Right and left lateral ventricles - Midline at level of the 3rd and 4th ventricles |
|  | **Brainstem/ Posterior fossa** | Trans-axial planes, probe placed at the sphenoidal fontanelle in transverse plane (right and left sides can be attempted) | Assess the midbrain, pons and cerebellar peduncles |
|  | **Cervical Spine** | Coronal plane, probe placed at the mastoid fontanelle in cranio-caudal orientation (right and left sides can be attempted) | Assess and obtain views of the cerebellum and cerebro-spinal junction |
|  | **Whole Spine**  **(image patient in prone position)** | Panoramic sagittal image or dual screen image of whole spine. | Document appearances of vertebral bodies and level of cord termination. If present, document presence of neural tube defect, cord truncation in caudal regression syndrome. |
|  |  | Sagittal image of the lumbar and sacral vertebrae | As above |
|  |  | Transverse images at level of cervical, thoracic and lumbar vertebrae | Demonstrate position of cord in transverse orientation, assess for any intraspinal lesions or diastematomyelia. |
|  |  | Optional: In smaller fetuses a coronal image of the spine is sometimes achievable. | Assess for same pathologies as per sagittal views described above |
|  |  |  |  |

**Table S2: Imaging Protocol for Perinatal Postmortem Ultrasound Study – Cardiothoracic System**

| **Body System:** | **Body Part:** | **Orientation:** | **Images Obtained & Assessed:** |
| --- | --- | --- | --- |
| **Thoracic** | **Lungs** | Sagittal views of both right and left lungs | Assess number of lobes of each lung and the presence/content of congenital diaphragmatic hernias or cystic pulmonary masses. |
| **Cardiac** | **Mediastinum/Heart** | Transverse section through the superior mediastinum/neck | Assess thymus, thyroid, trachea, carina and origin of great vessels  (i.e. aorta, superior vena cava, main pulmonary artery and branch pulmonary artery and patent ductus arteriosus). |
|  |  | Transverse section through the heart and great vessels | Assess for atrioventricular (AV) and ventriculo-arterial (VA) concordance and presence of septal defects |
|  |  | Sagittal section through the heart and great vessels | Obtain views of the ascending aorta, descending thoracic aorta and superior vena cava |

**Table S3: Imaging Protocol for Perinatal Postmortem Ultrasound Study – Abdominal System**

| **Body System:** | **Body Part:** | **Orientation:** | **Images Obtained & Assessed:** |
| --- | --- | --- | --- |
| **Abdominal** | **Liver/Biliary System** | Longitudinal sections | To be obtained through:   - Left lobe of liver - Left lobe of liver with caudate lobe - Liver with intrahepatic IVC - Liver mid-length with portal vein - Mid-section of right lobe of liver - Right lobe of liver with right kidney - Gallbladder - Common bile duct is rarely visible (calibre measurement usually not possible)   Assess for presence of biliary dilatation or masses, size and presence of gallbladder. |
|  |  | Transverse sections | To be obtained through:   - Right lobe of liver at liver dome - Right lobe of liver with right kidney - Left lobe of liver - Gallbladder (if present)   Assessment as above. |
|  | **Pancreas** | Transverse section of pancreas | Views should demonstrate head, body and tail of pancreas where possible. This organ is prone to early autolysis. |
|  | **Spleen** | Longitudinal section of spleen | Views of the spleen are notoriously difficult to obtain given relatively small size and similar echotexture to adjacent liver. Where enlarged, measurement should be taken as this could indicate underlying congenital metabolic disorder. |
|  |  | Transverse section of spleen |  |
|  | **Abdominal vessels** | Longitudinal section | Views obtained of:   - Abdominal aorta - IVC   Commonly, these vessels are thrombosed and echogenic. |
|  |  | Transverse views at level of SMA | Assess the anatomical orientation of SMA and SMV to assess for malrotation. This can be difficult however it is usually possible to visualise the retroperitoneal location of the duodenum. |
|  | **Kidneys**  **(image patient in both supine and prone positions)** | Longitudinal section of both kidneys and adrenal glands | Assess for presence of both kidneys (sometimes imaging in the pelvis is required to rule out pelvic kidney), presence of pelvicalyceal dilatation and renal cystic changes. It is also possible to visualise sequelae of adrenal haemorrhages or adrenal cysts, however these are rarely contributory to cause of death. |
|  |  | Transverse section of both kidneys and adrenal glands |  |
|  | **Bladder/ Pelvis/ Gonads** | Longitudinal sections | Views to include:   - Bladder - Uterus (where seen) - Testes (where seen, usually very small and can be beyond limits of imaging resolution)   Assess for features of bladder outlet obstruction, ureteric dilatation, presence of pelvic kidney. The uterus is usually well visualised in female subjects although ovaries are almost never visualised. |
|  |  | Transverse section of bladder |  |
|  | **Bowel** | Transverse section of all four abdominal quadrants | Assess for bowel thickening, pneumatosis, collections and large volume free fluid. |
